# Filamentation of asparagine synthetase in *Saccharomyces cerevisiae*

**DOI:** 10.1371/journal.pgen.1007737

**Published:** 2018-10-26

**Authors:** Shanshan Zhang, Kang Ding, Qing-Ji Shen, Suwen Zhao, Ji-Long Liu

**Affiliations:** 1 School of Life Science and Technology, ShanghaiTech University, Shanghai, China; 2 University of Chinese Academy of Sciences, Beijing, China; 3 Shanghai Institute of Biochemistry and Cell biology, Chinese Academy of Sciences, Shanghai, China; 4 iHuman Institute, ShanghaiTech University, Shanghai, China; 5 MRC Functional Genomics Unit, Department of Physiology, Anatomy and Genetics, University of Oxford, Oxford, United Kingdom; The University of North Carolina at Chapel Hill, UNITED STATES

## Abstract

Asparagine synthetase (ASNS) and CTP synthase (CTPS) are two metabolic enzymes crucial for glutamine homeostasis. A genome-wide screening in *Saccharomyces cerevisiae* reveal that both ASNS and CTPS form filamentous structures termed cytoophidia. Although CTPS cytoophidia were well documented in recent years, the filamentation of ASNS is less studied. Using the budding yeast as a model system, here we confirm that two ASNS proteins, Asn1 and Asn2, are capable of forming cytoophidia in diauxic and stationary phases. We find that glucose deprivation induces ASNS filament formation. Although ASNS and CTPS form distinct cytoophidia with different lengths, both structures locate adjacently to each other in most cells. Moreover, we demonstrate that the Asn1 cytoophidia colocalize with the Asn2 cytoophidia, while Asn2 filament assembly is largely dependent on Asn1. In addition, we are able to alter Asn1 filamentation by mutagenizing key sites on the dimer interface. Finally, we show that *ASN1*^*D330V*^ promotes filamentation. The *ASN1*^*D330V*^ mutation impedes cell growth in an *ASN2* knockout background, while growing normally in an *ASN2* wild-type background. Together, this study reveals a connection between ASNS and CTPS cytoophidia and the differential filament-forming capability between two ASNS paralogs.

## Author summary

Asparagine synthetase (ASNS) is an essential enzyme for biosynthesis of asparagine. We have recently shown that ASNS, similar to CTP synthase (CTPS), can assemble into snake-shaped structures termed cytoophidia. In this study, we reveal that the ASNS cytoophidium stays close with the CTPS cytoophidium in most cells. Two ASNS proteins, Asn1 and Asn2, localize in the same structure. The Asn1 protein is important for the formation of the Asn2 filaments. Mutant cells with branching Asn1 cytoophidia grow slower than wild-type cells. Our findings provide a better understanding of the ASNS cytoophidium as well as its relationship with the CTPS cytoophidium.

## Introduction

Intracellular compartmentation is crucial for the function of a cell. In 2010, three studies reported that the metabolic enzyme CTP synthase (CTPS), forms filamentous compartments, termed cytoophidia, in fruit flies, bacteria and budding yeast cells [1–3]. Subsequent studies revealed that the CTPS cytoophidium also exists in fission yeast, human and *Arabidopsis* cells [4–7]. CTPS can form cytoophidia not only in the cytoplasm but also in the nucleus of eukaryotic cells [8–10].

A genome-wide screening identified that at least 23 proteins, including CTPS and asparagine synthetase (ASNS), can form filaments in budding yeast [9]. Both CTPS and ASNS are glutamine-utilizing enzymes. While CTPS converts the nucleotide UTP into CTP, the enzyme ASNS catalyzes the conversion of L-aspartate into L-asparagine. Both enzymes have a significant impact on glutamine homeostasis [11, 12].

In *Saccharomyces cerevisiae*, there are two *ASNS* genes, *ASN1* and *ASN2*, and two *CTPS* genes, *URA7* and *URA8* [13, 14]. Genetic studies have demonstrated that asparagine auxotrophy in yeast results from a combination of *ASN1* and *ASN2* mutations, while neither *ASN1* nor *ASN2* mutation can individually lead to total auxotrophy [15]. Double *ASN1* and *ASN2* mutants have no effect on cell cycle progression in *S*. *cerevisiae*, while *ASNS* mutation lead to G1 phase arrest in hamster [13, 16]. *ASNS* knockdown significantly deregulated the expression of CDK4, CDK6 and Cyclin D1 and suppressed the growth of melanoma cells and epidermoid carcinoma cells [17].

To better understand filamentation of metabolic enzymes, here we use ASNS and CTPS in *S*. *cerevisiae* as examples. We are particularly interested in the following important questions: 1) Is ASNS filamentation sensitive to glucose deprivation? 2) Is ASNS present in the same cytoophidium as CTPS? 3) Does the Asn1 cytoophidium behave identically to the Asn2 cytoophidium? 4) What are the consequences when the ASNS filamentation is disturbed?

## Results

### ASNS can form cytoophidia in *S*. *cerevisiae*

**Fig 1:**
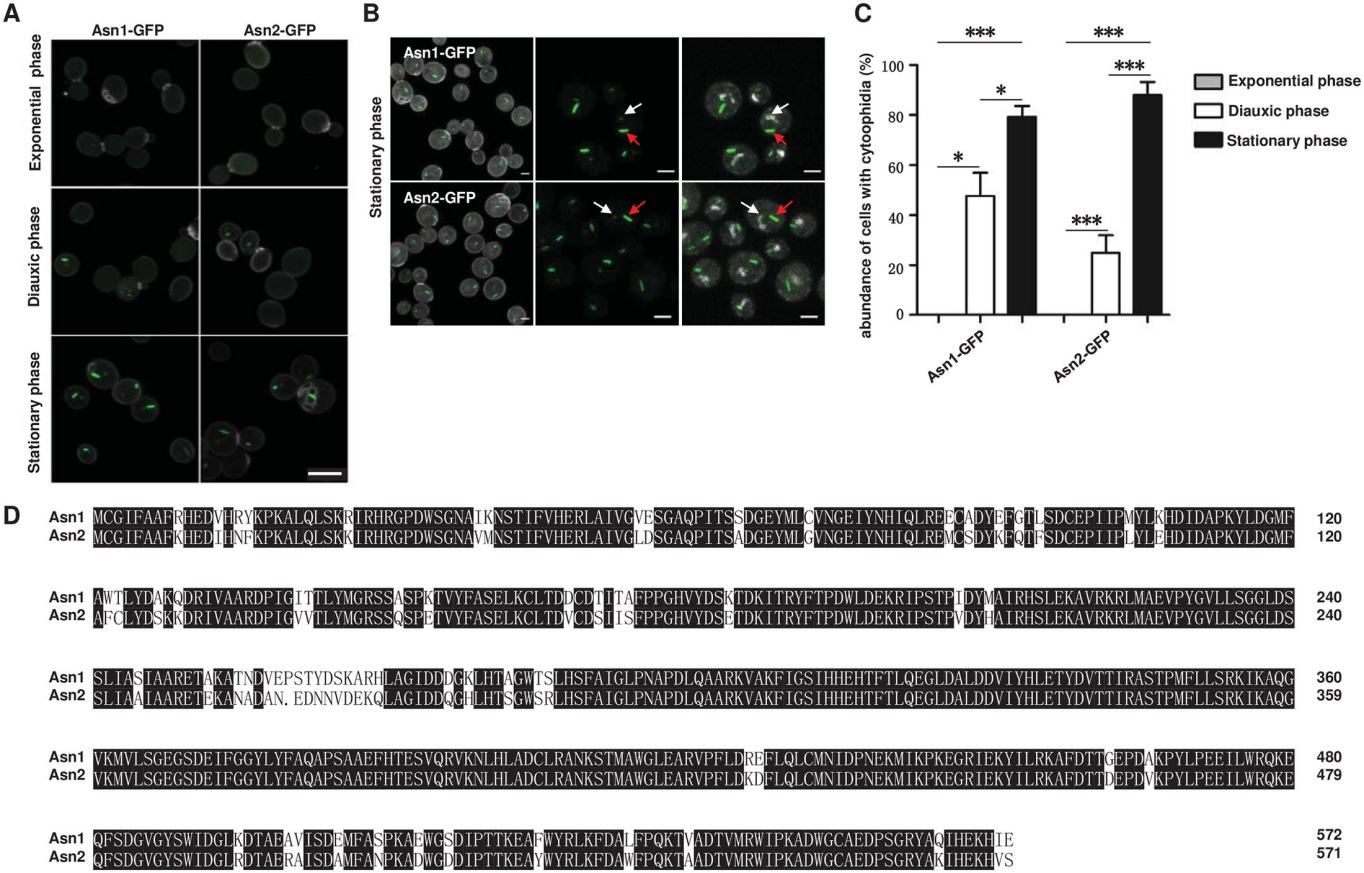
Asn1 and Asn2 form filaments in *S*. *cerevisiae*. (A) Confocal images of yeast cells showing the filamentation capacity of Asn1 and Asn2 in exponential, diauxic and stationary phase. Scale bar, 5 μm. Asn1 and Asn2 are marked by GFP. Cell wall is stained by Calcofluor White stain. (B) In stationary phase, both Asn1 and Asn2 form cytoophidia in the cytoplasm and nucleus. Scale bar, 2 μm. The nuclei are labelled with DAPI. White arrows indicate nuclear cytoophidia and red arrows indicate cytoplasmic cytoophidia. (C) Quantification of the cells containing visible cytoophidia in the exponential, diauxic and stationary phase. *P<0.05 and ***P<0.0001. Error bars show SEM. (D) The amino acid sequence alignment of Asn1 and Asn2.

ASNS has been discovered to form cytoophidia in the cytoplasm and nucleus *in vivo* [9]. Using GFP-tagged strains, we confirmed that both Asn1 and Asn2 could form cytoophidia in the cytoplasm and nucleus (Fig 1A and 1B). Furthermore, the GFP tagged Asn1 or Asn2 cells were cultured in different growth phases to test the cytoophidium abundance. Previous studies defined the different growth phases of budding yeast [18, 19]. We collected a population of cells cultured for 6 hours, 24 hours and 7 days. These three durations represent the exponential, diauxic and stationary phases, respectively (Fig 1A–1C). In the exponential phase, neither Asn1 nor Asn2 formed detectable cytoophidia under light microscopy. In contrast, we could detect Asn1 and Asn2 cytoophidia in the diauxic phase. The percentage of cells containing cytoophidia was 35.97% (Asn1) and 18.84% (Asn2), respectively. In the stationary phase, 80.69% of cells showed Asn1 cytoophidia and 90.82% cells contained Asn2 cytoophidia. After transitioning from the exponential phase to the stationary phase, it was clear that the abundances of both Asn1 and Asn2 cytoophidia were greatly increased (Fig 1C). The alignment of the Asn1 and Asn2 proteins shows 88% identity (Fig 1D). These results imply that the formation of cytoophidia correlates with the growth phases and the cytoophidium responds to the change of culture condition. These data confirm our previous study in which we screened 4159 GFP-tagged proteins in budding yeast [9].

### Glucose deprivation induces ASNS cytoophidium formation

CTPS has been found to form filaments due to the deprivation of carbon source [3]. Among five filament-forming enzymes (Gcd2-GFP, Glt1-GFP, Psa1-GFP, Sui2-GFP and Ura7-GFP), only Ura7 reacted to carbon deprivation. To test whether carbon deprivation regulation is a CTP synthase-exclusive process or a more general process, we conducted the glucose deprivation experiment in Asn1-GFP and Asn2-GFP cells.

**Fig 2:**
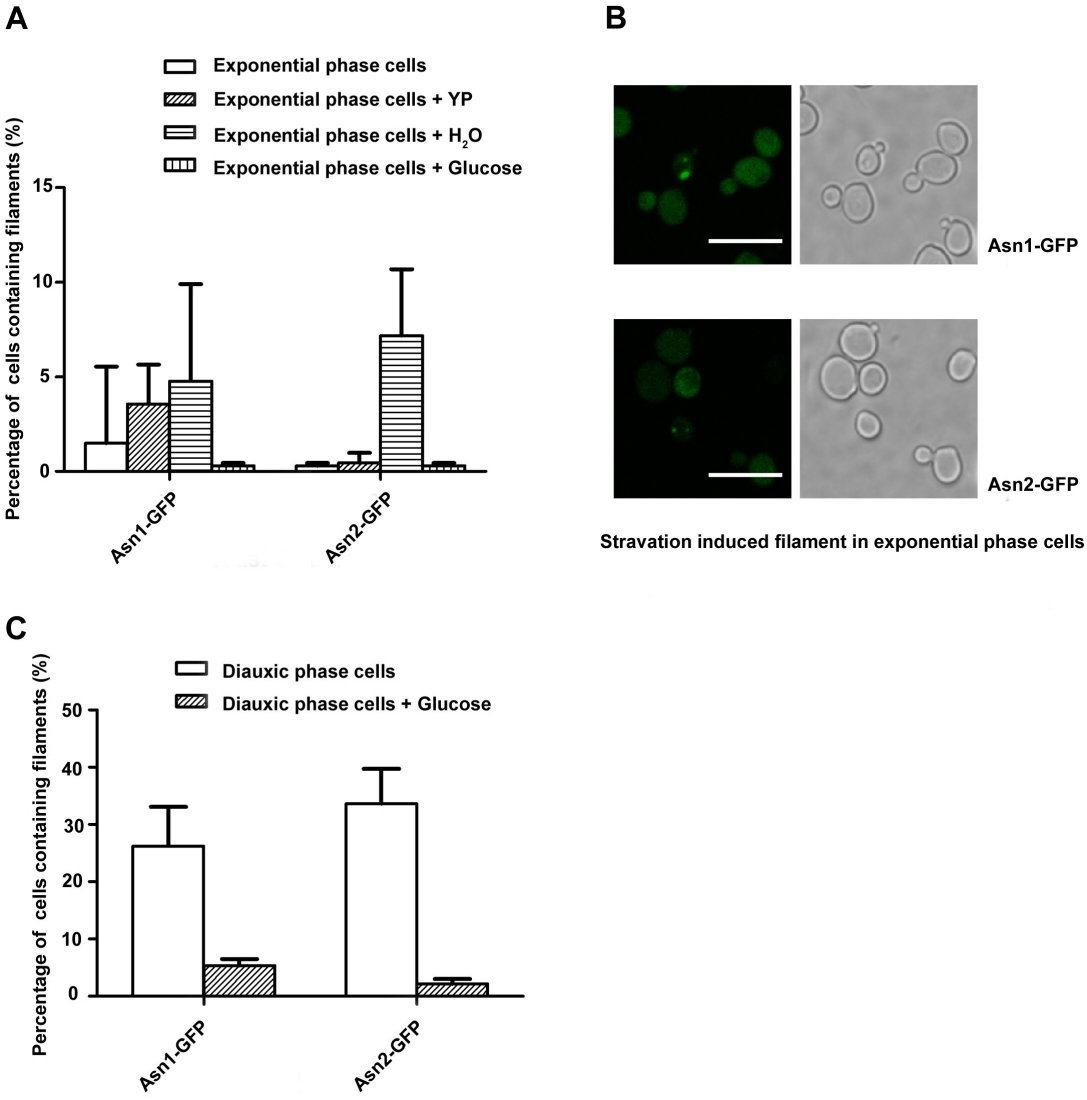
Filament formation regulated by carbon nutrient starvation. (A, B) deprivation of glucose in media induces Asn1 and Asn2 cytoophidia in the exponential phase. (C) The addition of glucose at the diauxic shift causes Asn1 and Asn2 cytoophidia to disassemble. Error bars show 95% confidence interval. Scale bars, 10 μm. YP stands for yeast extract peptone medium without dextrose.

Results indicate that ASNS based filaments show similar behavior to CTP synthase-based filaments under carbon source starvation. Distilled water treatment induced filament formation in both Asn1-GFP and Asn2-GFP strains and YP media induced filaments in the Asn1-GFP strain (Fig 2A and 2B). The add-in of glucose showed a severe inhibition effect to those starvation-induced filaments in both strains. Asn1 and Asn2 filament positive cells percentages were merely around 10%, far less than the percentage of Asn1 and Asn2 filaments when the strains reach the stationary phase (Fig 2A). At the diauxic shift, when cells begin to utilize other carbon sources rather than glucose, the add-in of glucose pulls cells back to glucose metabolism. Results showed that the add-in of glucose severely reduced filaments in both Asn1-GFP and Asn2-GFP strains (Fig 2C).

### Spatial association of ASNS cytoophidia and CTPS cytoophidia

**Fig 3:**
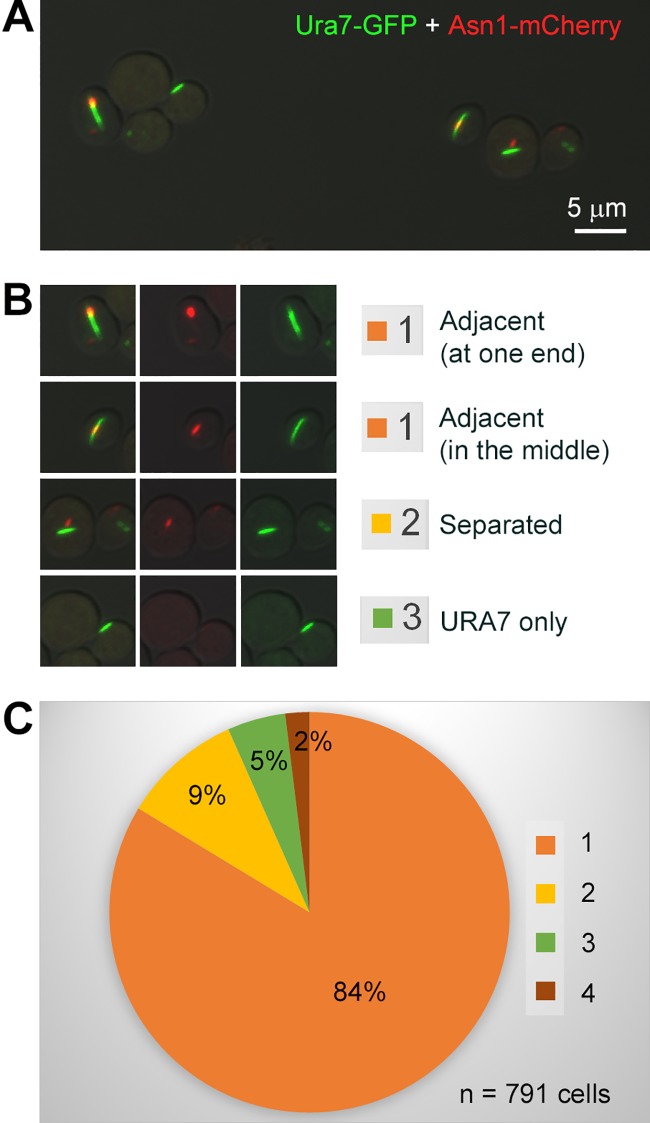
Association of ASNS cytoophidia and CTPS cytoophidia. (A) Spatial relationship of ASNS cytoophidia and CTPS cytoophidia in stationary phase. Ura7 is shown in green and Asn1 is shown in red. Scale bar, 5 μm. (B) Representative images showing four kinds of spatial relationship between ASNS cytoophidia and CTPS cytoophidia. (C) Quantification of the two cytoophidia spatial relationship.

ASNS is responsible for the biosynthesis of aspargine, while CTPS catalyzes the rate-limiting reaction for the de novo synthesis of the pyrimidine nucleotide CTP. In budding yeast, both ASNS and CTPS use glutamine as the substrate and both enzymes have the ability to form cytoophidia. To study the relationship between CTPS and ASNS, we co-expressed GFP-tagged Ura7 (CTPS) and mCherry-tagged Asn1 (ASNS) in the same strain. We classified the spatial relationship of the Ura7 cytoophidium and the Asn1 cytoophidium into the following four categories. 1) Adjacent (The Ura7 cytoophidium localizes adjacently to the Asn1 cytoophidium, either head-to-head or side-by-side). 2) Separated (The Ura7 cytoophidium and the Asn1 cytoophidium localize separately with each other). 3) Ura7 only (Only Ura7-GFP is detectable). 4) Asn1 only (Only Asn1-mCherry is detectable) (Fig 3). Among 791 fluorescence positive cells growing at the stationary phase, 84% fell into the “Adjacent” category, while only 9% cells showed separated distributions of Asn1 and Ura7 (Fig 3C). Moreover, very few cells showed only one type of cytoophidia (5% Ura7 only and 2% Asn1 only, respectively).

**Fig 4:**
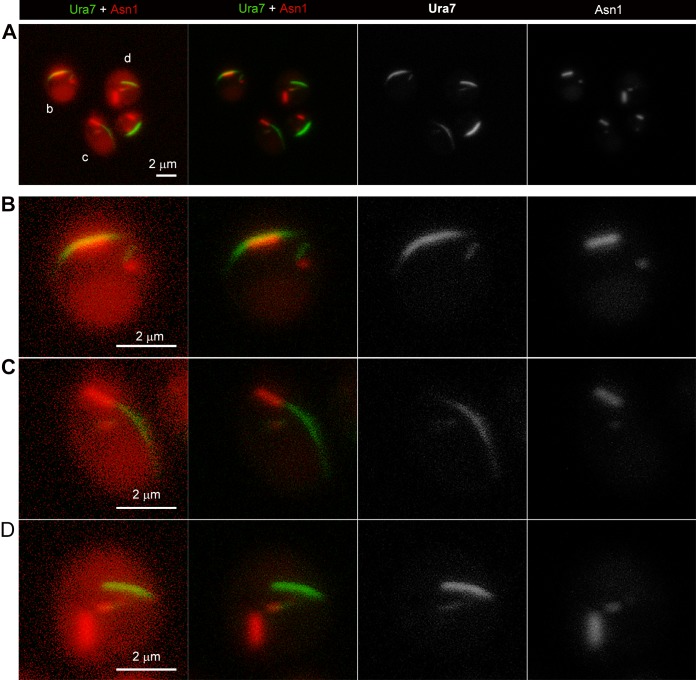
Magnified images of ASNS cytoophidia and CTPS cytoophidia in cytoplasm and nucleus. (A) ASNS cytoophidia and CTPS cytoophidia in nucleus have similar association pattern as in cytoplasm. Scale bar, 2 μm. (B-D) High magnification of yeast cells show that the spatial relationship of ASNS cytoophidia and CTPS cytoophidia in nucleus. Scale bar, 2 μm.

CTPS and ASNS can form cytoophidia both in the cytoplasm and in the nucleus. Nuclear cytoophidia are much smaller and more difficult to be detected than cytoplasmic cytoophidia. The data in Fig 3 were mostly focusing on the cytoplasmic cytoophidia. To determine the relationship of nuclear cytoophidia between Ura7 and Asn1, we captured many cells under high resolution. In most cases, one nucleus contains one Ura7 positive cytoophidium and one Asn1 positive cytoophidium. The Asn1 nuclear cytoophidium seemed always localizing adjacently to the Ura7 nuclear cytoophidium, no matter whether the Asn1 cytoplasmic cytoophidium is adjacent to or separated from the Ura7 cytoplasmic cytoophidium in the same cell (Fig 4). The intimate spatial relationship both in the cytoplasm and in the nucleus suggest a functional coordination between ASNS and CTPS cytoophidia. Further studies are required for understanding the coordinated filamentation between these two metabolic enzymes.

### Asn1 cytoophidia colocalize with Asn2 cytoophidia

**Fig 5:**
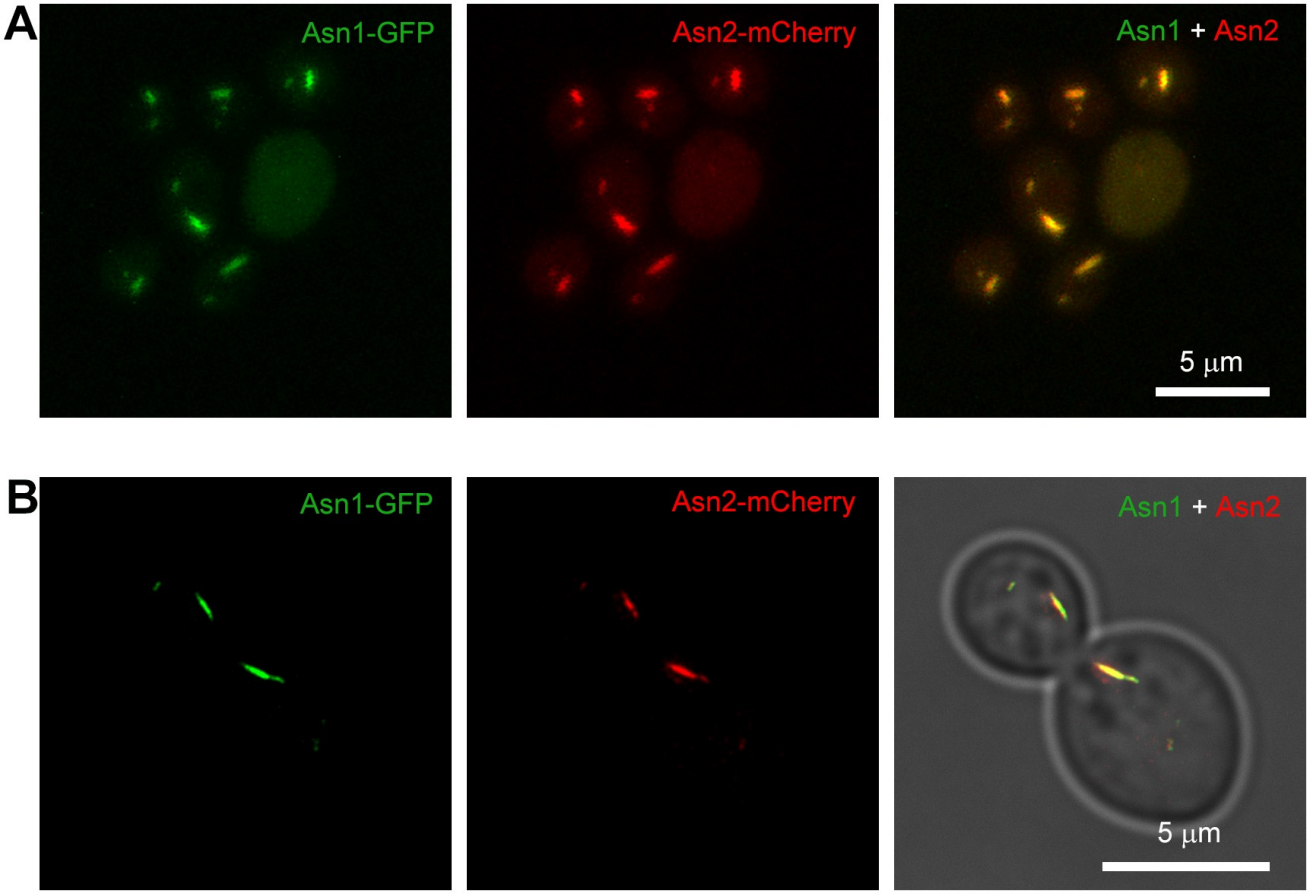
Asn1 cytoophidium colocalizes with Asn2 cytoophidium. Confocal images (A) and STED images (B) of yeast cells displayingthe colocalization of Asn1 cytoophidium and Asn2 cytoophidium. Scale bar, 5 μm. ASN1 is tagged with GFP and ASN2 is tagged with mCherry. Double tagged yeast cells are collcected in stationary phase.

Disrupting either *ASN1* or *ASN2* alone has no effect on growth rate but simultaneous disruption of *ASN1* and *ASN2* leads to an asparagine auxotroph mutant [15]. Since both Asn1 and Asn2 formed cytoophidia and they had high identity, we were curious about the subcellular localization of Asn1 and Asn2 cytoophidia. To this end, we tagged Asn1 with GFP and tagged Asn2 with mCherry to obtain a double-labelling strain. Confocal images of double-labelling cells showed that Asn1 cytoophidia colocalize with Asn2 cytoophidia both in the cytoplasm and in the nucleus (Fig 5A). Super-resolution images that were obtained under stimulated emission depletion (STED) microscopy confirmed the colocalization of Asn1 and Asn2 cytoophidia (Fig 5B).

### Asn1 is influential for the filamentation of Asn2

**Fig 6:**
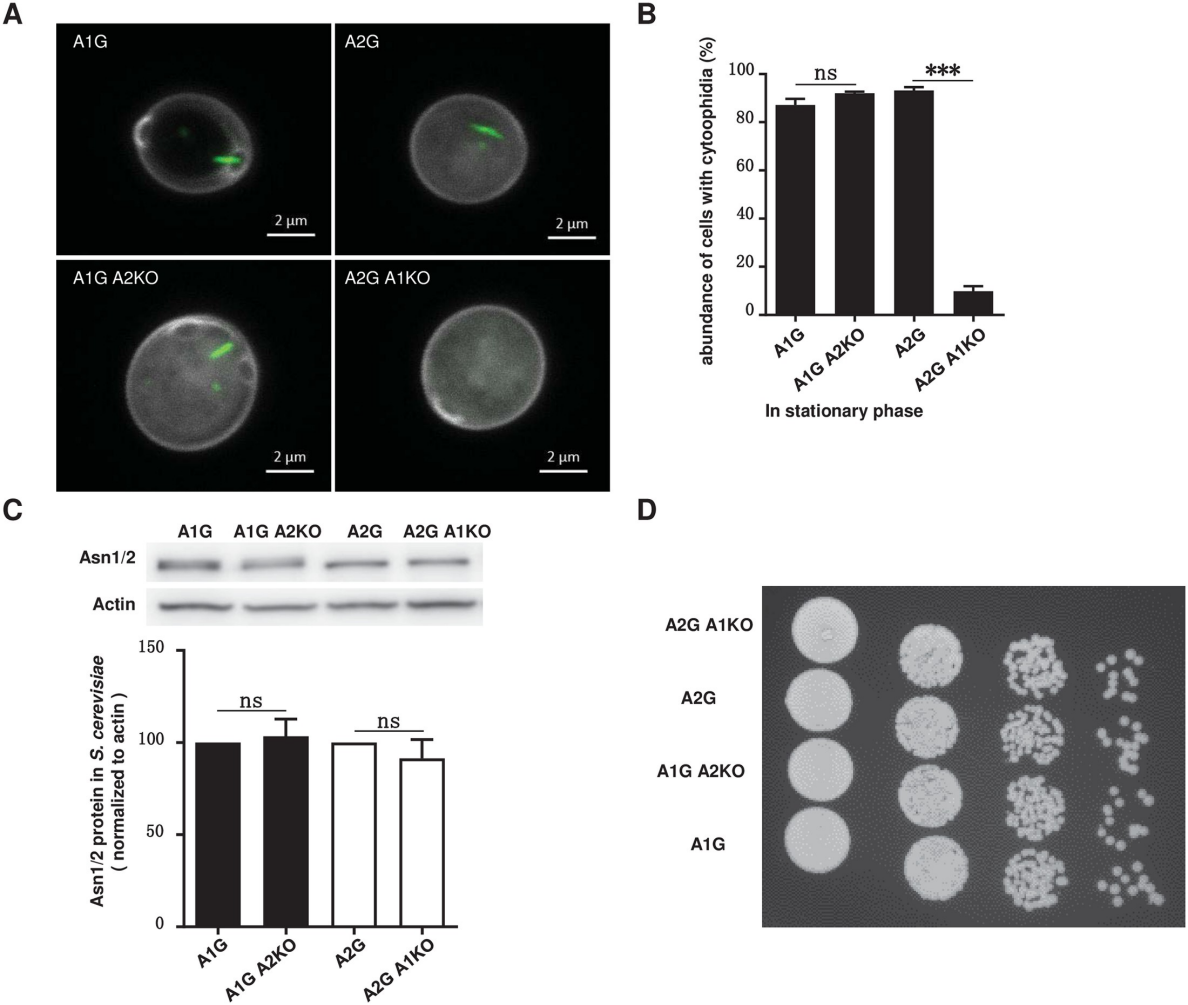
Asn2 cytoophidium is dependent on Asn1. (A) Confocal images of cytoophidium in A1G, A2G, A1G A2KO, A2G A1KO cells. Scale bar, 2 μm. (B) Quantification of cytoophidium abundance in A1G, A2G, A1G A2KO, A2G A1KO cells showing that Asn1 is crucial for Asn2 cytoophidium formation. ***P<0.0001. n.s. = not significant. Error bars show SEM. (C) Western blot analysis of ASNS protein level and quantification of the relative ASNS abundance in A1G, A2G, A1G A2KO, A2G A1KO cells. n.s. = not significant. Error bars show SEM. (D) Spot assay of A1G, A2G, A1G A2KO and A2G A1KO cells. Eight microliters of four 10-fold serial dilution of each yeast culture was spotted.

Next, we wanted to know whether filamentation of Asn1 and Asn2 is independent with each other. Our strategy was to knockout one *ASNS* and then tag the other ASNS protein with GFP. We generated the following four strains: 1) Asn1-GFP (A1G), 2) Asn2-GFP (A2G), 3) Asn1-GFP Asn2-knockout (A1G A2KO), and 4) Asn2-GFP Asn1-knockout (A2G A1KO). We checked the morphology and measured the abundance of the ASNS cytoophidia. ASNS cytoophidia were detectable in most A1G, A2G and A1G A2KO cells, but only very few A2G A1KO cells showed ASNS cytoophidia (Fig 6A). Whilst only 9.85% of the A2G A1KO cells showed the Asn2 cytoophidium, more than 80% of the A2G cells contain the Asn2 cytoophidium (Fig 6B). These results make us believe that Asn1 has a great impact on the filamentation of Asn2. In the A1G and A1G A2 KO cells, the abundances of ASNS cytoophidia reached 87.12% and 92.01%, respectively (Fig 6B). In addition, we compared the ASNS protein level and growth rate among these four strains. ASNS protein levels showed no significant difference among all four strains (Fig 6C). To study whether the *ASNS* knockout has an effect on cell growth, we did spot assay experiments to test the cell growth rate. Our results showed that neither *ASN1* knockout nor *ASN2* knockout alone affected cell growth rate (Fig 6D).

### Dimerization interface of Asn1

The biosynthesis of asparagine from aspartic acid occurs in three steps: activation of aspartate, glutamine hydrolysis, and synthesis of a beta-aspartyl-AMP intermediate and its subsequent reaction with ammonia [20]. A study of *E*. *coli* ASNS B (ASNB) showed that ASNS consists of two distinct domains: an N-terminal domain that mediates the hydrolysis of glutamine to glutamate and a C-terminal domain that is involved in the activation of aspartate by ATP [20]. The dimeric interface of *E*. *coli* ASNB is formed by both the N- and C-terminal region of each monomer, which includes an α-helix-turn-β-sheet structure (A13 –S40) of the N-terminal and an α-helix-turn-α-helix structure (V301 –M339) of the C-terminal domain, with both ligand-binding sites being distant from the dimer interface. Several salt-bridge pairs enhance the binding between two dimers, such as R17:D306, R25:D310, R28:D30, E48:R334 in ASNB. In addition, R25 and R334 could potentially form a cation-π interaction with Y313 and W34 respectively.

**Fig 7:**
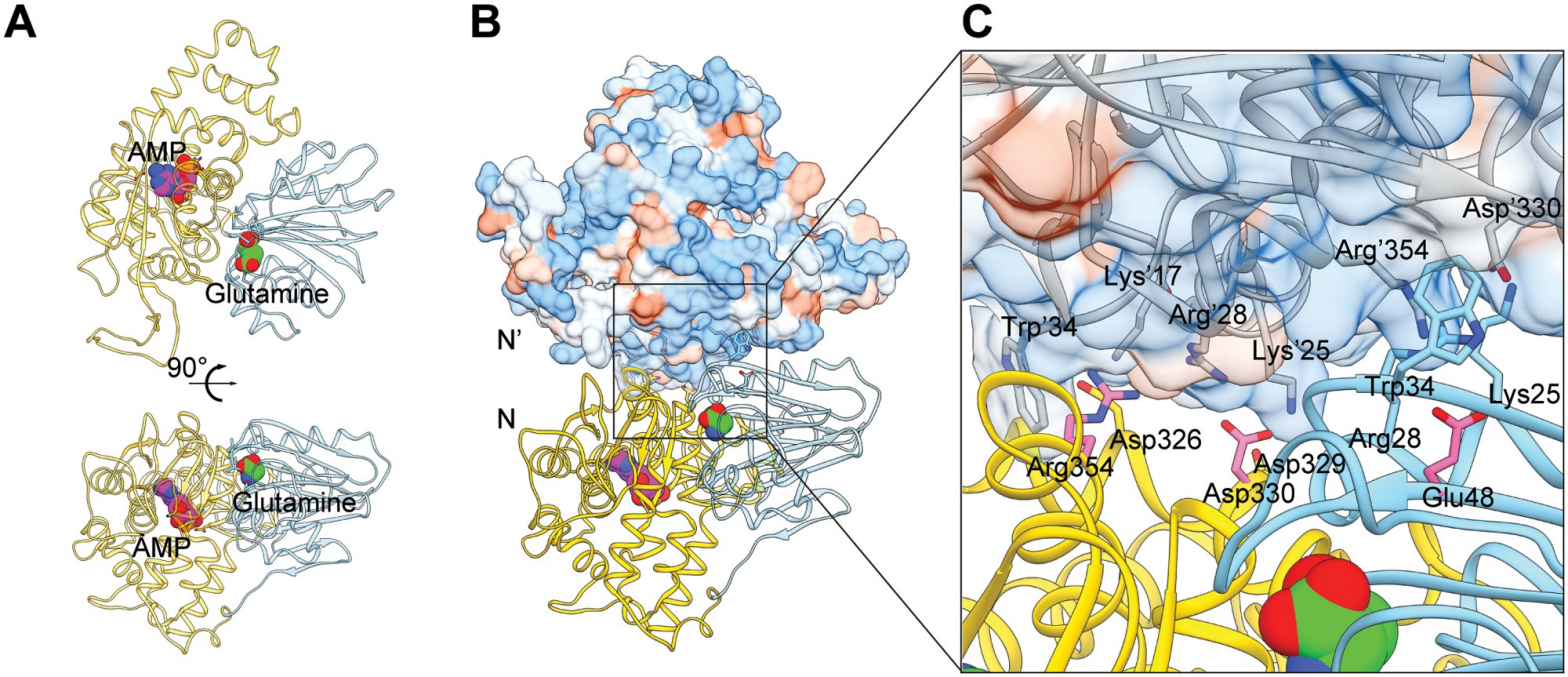
Homology model of Asn1 and its dimer. (A) Homology model of Asn1 monomer using *E*.*coil* ASNB as reference. N-terminal domain containing the glutamine binding site and C-terminal domain containing the AMP binding site are shown in cyan and yellow, respectively. Glutamine is shown in green and AMP in magenta. (B) Homology model of Asn1 dimer. N-N’ presents Asn1 dimer interface. N’ is shown as hydrophobicity surface view. Blue represents the hydrophilic surface area and orange represents the hydrophobic surface area. (C) Asn1 dimer interface. Key residues in protein are labelled and mutation sites may have a strong effect on dimerization of Asn1 are highlighted with pink (D330, R354, E48).

The homology model of Asn1 shared the same dimer interface and conserved salt-bridge interactions with the structure of *E*. *coli* ASNB, including K25:D330, R28:D330, E48:R354, as in Asn1 (Fig 7). K25 and R354 also formed cation-π interactions with Y333 and W34, respectively. We chose D330V, R354E and E48K for the following research (Fig 7B and 7C), aiming to disrupt or decrease the dimerization thus filament formation.

### Disrupting dimerization of Asn1 affects its filamentation

We utilized a site-directed mutagenesis strategy to target the specific site on Asn1 dimerization interface. To avoid the complementary effect of Asn1 and Asn2, we knocked out Asn2 in order to study the Asn1 cytoophidium only. We first generated three GFP tagged Asn1 mutant strains (D330V, R354E and E48K). Then we knocked out *ASN2* in each of these three *ASN1* mutant strains. Eventually we obtained the following eight strains: ASN1^WT^-GFP ASN2 WT, ASN1^D330V^-GFP ASN2 WT, ASN1^R354E^-GFP ASN2 WT, ASN1^E48K^-GFP ASN2 WT, ASN1^WT^-GFP ASN2 KO, ASN1^D330V^-GFP ASN2 KO, ASN1^R354E^-GFP ASN2 KO and ASN1^E48K^-GFP ASN2 KO.

**Fig 8:**
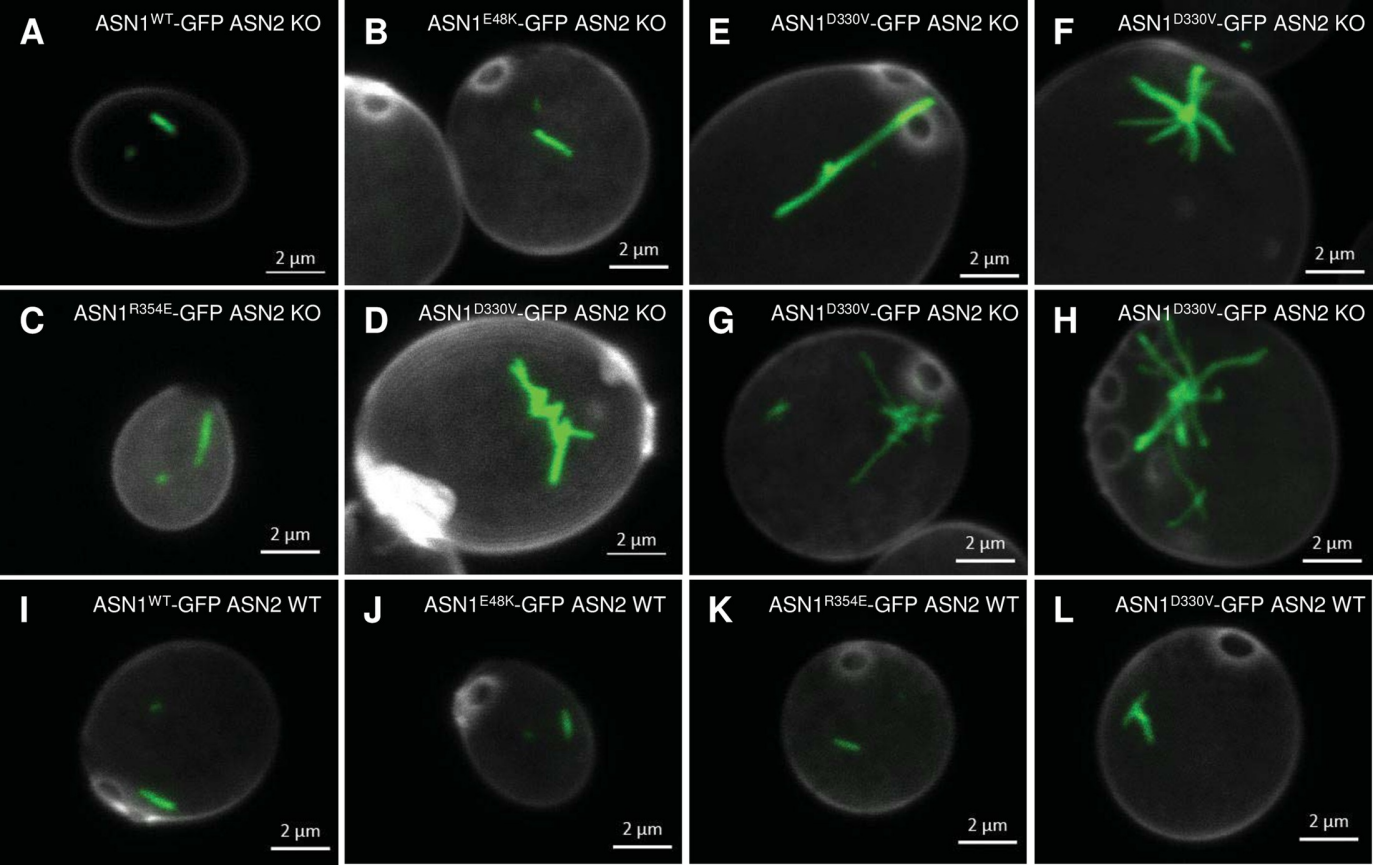
Cytoophidia in Asn1 mutants. (A-D) Morphology of Asn1^WT^, Asn1^E48K^, Asn1^R354E^ and Asn1^D330V^ cytoophidium in ASN2 knockout background. (E-H) Varied morphology of Asn1^D330V^ cytoophidium in ASN1^D330V^-GFP ASN2 KO cells. (I-L) Morphology of Asn1^WT^, Asn1^E48K^, Asn1^R354E^ and Asn1^D330V^ cytoophidium in ASN2 wild type background. Scale bar, 2 μm.

Similar to ASN1^WT^, both ASN1^E48K^ and ASN1^R354E^ showed one large cytoplasmic cytoophidium and one small nuclear cytoophidium in ASN2 knockout background (Fig 8A, 8B and 8C). A typical cytoplasmic cytoophidium is straight without any branches or slightly curved. However, the Asn1 cytoophidium in ASN1^D330V^-GFP ASN2 KO cells displayed irregular morphology with a variety of shapes, many of which contain multiple branches (Fig 8D–8H). In ASN2 wild type background, ASN1^WT^, ASN1^R354E^ and ASN1^E48K^ present filament structure (Fig 8I–8K). Whereas the morphology change of ASN1^D330V^ cytoophidium in ASN1^D330V^-GFP ASN2 WT cells is less dramatic (Fig 8L).

**Fig 9:**
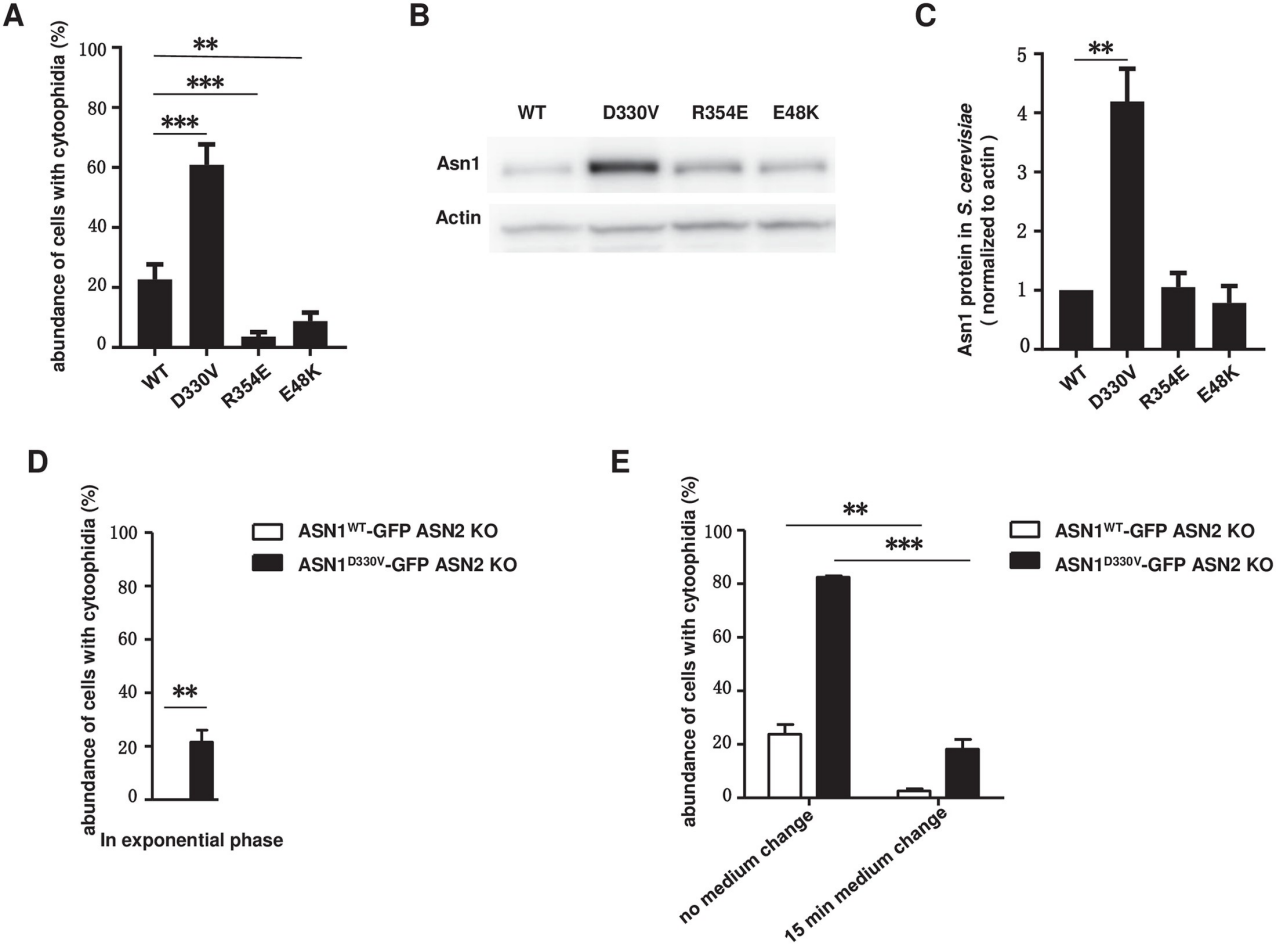
Asn1 cytoophidium abundance and relative protein levels are altered in D330V mutant. (A) The proportion of cells with Asn1 cytoophidia in different dimer interface mutants. **P<0.001. ***P<0.0001. Error bars show SEM. B) Western blot analysis of Asn1 relative protein levels. (C) Fold change of Asn1 protein level in all mutants. **P<0.001. Error bars show SEM. (D) Quantification of cytoophidium abundance of ASN1^WT^-GFP ASN2 KO cells and ASN1^D330V^-GFP ASN2 KO cells in exponential phase. **P<0.001. Error bars show SEM. (E) Effect of 15 min medium change on ASN1^WT^-GFP ASN2 KO cells and ASN1^D330V^-GFP ASN2 KO cells. **P<0.001. ***P<0.0001. Error bars show SEM.

Furthermore, we checked the percentage of cells containing cytoophidia and the Asn1 protein level in the stationary phase. In the ASN1^WT^-GFP ASN2 KO strain, the percentage of cells with cytoophidia reached 22.72%. When we cultured mutant strains in the same medium, 60.91% of ASN1^D330V^-GFP ASN2 KO cells showed obvious Asn1 cytoophidia in the stationary phase, while only 3.65% and 8.81% of ASN1^R354E^-GFP ASN2 KO cells and ASN1^E48K^-GFP ASN2 KO cells, respectively, contained Asn1 cytoophidia (Fig 9A). The protein level of Asn1 in ASN1^D330V^-GFP ASN2 KO cells was upregulated significantly comparing with the WT control and the other two mutants (R354E and E48K) (Fig 9B and 9C).

### *ASN1*^*D330V*^ promotes filamentation

In ASN1-GFP cells, we have already shown that there are no visible Asn1 cytoophidia in the exponential phase (Fig 1B). To investigate whether the Asn1^D330V^ had a similar behavior to Asn1^WT^, we collected ASN1^WT^-GFP ASN2 KO cells and ASN1^D330V^-GFP ASN2 KO cells after a 6-hour culture. In the ASN1^WT^-GFP ASN2 KO cells, the Asn1 cytoophidium was still undetectable in the exponential phase. To our surprise, 21.58% of ASN1^D330V^-GFP ASN2 KO cells showed visible Asn1 cytoophidia (Fig 9D). These results suggest that D330V promotes the assembly of Asn1 cytoophidia.

To determine if the assembly of ASNS cytoophidia is sensitive to nutrient availability, we cultured both ASN1^WT^-GFP ASN2 KO cells and ASN1^D330V^-GFP ASN2 KO cells both for 10 days and then switched them to fresh media for 15 minutes. The percentage of cells containing cytoophidia dropped from 23.76% to 2.63% in the ASN1^WT^-GFP ASN2 KO strain, while decreasing from 82.71% to 18.21% in the ASN1^D330V^-GFP ASN2 KO strain (Fig 9E). In conclusion, both Asn1^WT^ and Asn1^D330V^ cytoophidia are sensitive to culture conditions.

### The *ASN1*^*D330V*^ cells grow slowly in the absence of *ASN2*

**Fig 10:**
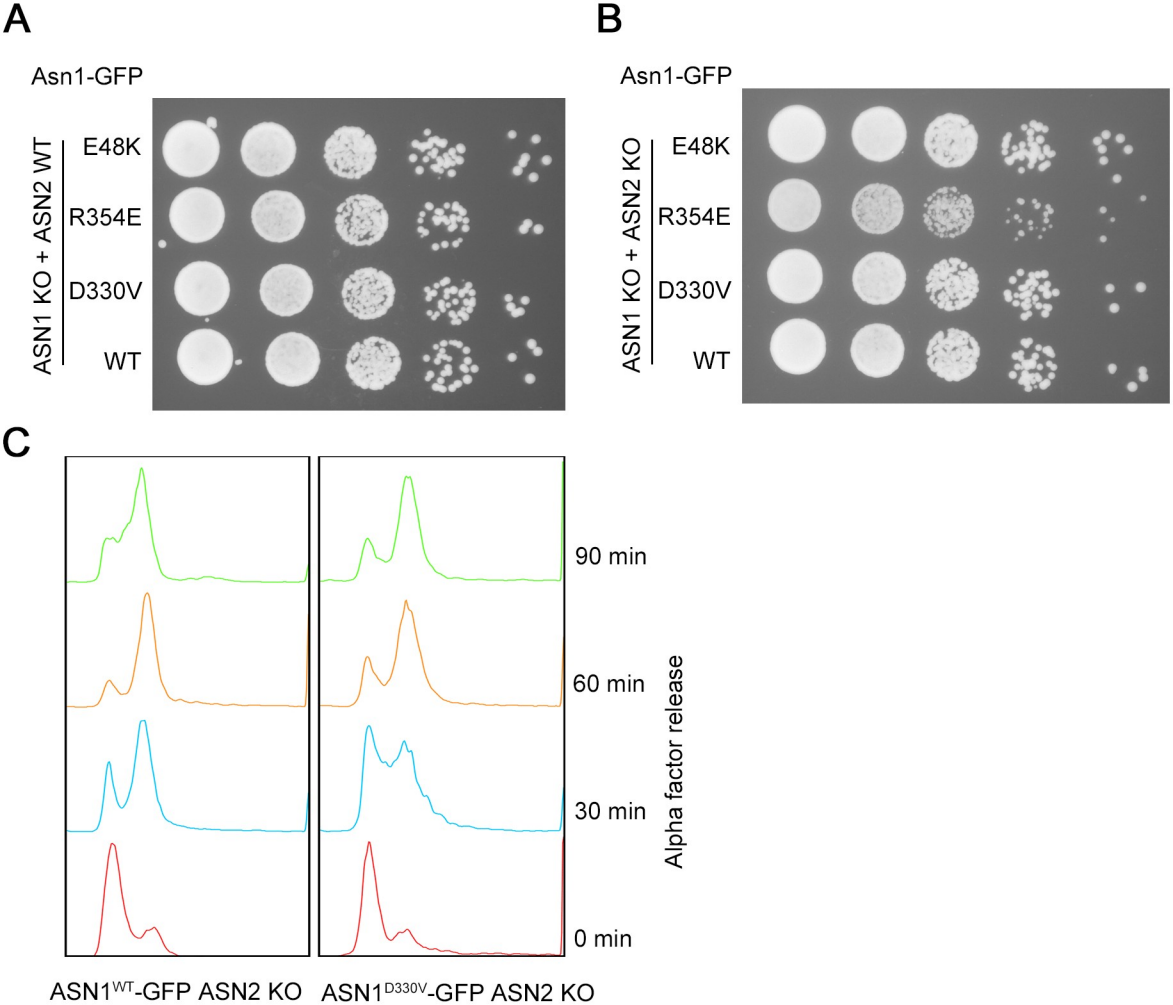
ASN1^D330V^-GFP ASN2 KO cells show lower growth rate and slower cell cycle progression. (A) Spot assay of mutants before ASN2 knockout (ASN1^WT^-GFP ASN2 WT, ASN1^D330V^-GFP ASN2 WT, ASN1^R354E^-GFP ASN2 WT and ASN1^E48K^-GFP ASN2 WT). (B) Spot assay of mutants after ASN2 knockout (ASN1^WT^-GFP ASN2 KO, ASN1^D330V^-GFP ASN2 KO, ASN1^R354E^-GFP ASN2 KO and ASN1^E48K^-GFP ASN2 KO). (C) FACS analysis of ASN1^WT^-GFP ASN2 KO cells and ASN1^D330V^-GFP ASN2 KO cells.

We used spot assays to compare the growth rates of various strains. In a normal *ASN2* background, the growth rate of ASN1^D330V^-GFP cells was similar to that of ASN1^WT^-GFP and of the other two mutants, ASN1^R354E^-GFP and ASN1^E48K^-GFP (Fig 10A). Both ASN1^R354E^-GFP and ASN1^E48K^-GFP cells grew normally in an ASN2 KO background. However, the growth of ASN1^D330V^-GFP cells slowed down when *ASN2* was knockout (Fig 10B).

Next, we used the alpha mating factor to arrest the ASN1^WT^-GFP ASN2 KO cells and ASN1^D330V^-GFP ASN2 KO cells in G1 phase, and subsequently released those cells at the same time. We collected those groups at 0 min, 30 min, 60 min and 90 min after alpha factor release and analyzed the cell cycle by FACS (Fig 10C). At 30 min after release, there were two distinct peaks formed by ASN1^WT^-GFP ASN2 KO cells and more than a half of cells successfully progressed from the G1 to G2 phase. However, ASN1^D330V^-GFP ASN2 KO cells delayed the transition from G1 to G2 phase. At 60 min after the release, the proportion of ASN1^WT^-GFP ASN2 KO cells in G1 phase was obviously lower than that of ASN1^D330V^-GFP ASN2 KO cells. These results suggest that the *ASN1*^*D330V*^ mutation impairs cell growth in the absence of *ASN2*.

## Discussion

The two CTPS proteins, Ura7 and Ura8, colocalize with each other in *S*. *cerevisiae* [3]. Like CTPS proteins, the two ASNS proteins, Asn1 and Asn2, are capable of assembling into cytoophidia in the cytoplasm and the nucleus. Looking into the relationship between CTPS cytoophidia and ASNS cytoophidia, we find the following interesting features. 1) ASNS cytoophidia do not colocalize with CTPS cytoophidia, suggesting ASNS and CTPS form different structures. 2) Both CTPS and ASNS use glutamine as a substrate in budding yeast. 3) Both CTPS and ASNS form cytoophidia not only in the cytoplasm, but also in the nucleus. 4) The number of ASNS cytoophida, similar to that of CTPS cytoophidia, is one in the cytoplasm and one in the nucleus in many cells. 5) In most budding yeast cells that we analyzed, the ASNS cytoophidia localize adjacently to the CTPS cytoophidia. These two types of structures can touch with each other head-to-head or side-by-side. 6) The close spatial relationship between ASNS and CTPS does not limited to the cytoplasm. In the nucleus, we often observe that the ASNS filament sits next to the CTPS filament. 7) The length of the ASNS cytoplasmic cytoophidium is shorter than that of the CTPS cytoplasmic cytoophidium. 8) The length of the ASNS nuclear cytoophidium is shorter than that of the CTPS nuclear cytoophidium. 9) Nuclear cytoophidia are much smaller than cytoplasmic cytoophidia, whether the composition is CTPS or ASNS. 10) Both CTPS and ASNS cytoophidia are very sensitive to nutrient changes and growth phases. Further studies are required to understand the dynamic interaction and functional connection, if any, between ASNS and CTPS cytoophidia. Previous studies have shown that CTPS cytoophidia have interfilament interaction with inosine monophosphate dehydrogenase (IMPDH) cytoophidia [21, 22], suggesting that the interaction between two cytoophidia may be a phenomenon more general than we have appreciated.

Asn1 and Asn2 have identical distribution pattern, raising the possibility that Asn1 and Asn2 have the same filament-forming capability. To our surprise, our data indicate that Asn1 and Asn2 behave distinctly in the absence of its partner. Removing Asn2 does not prevent Asn1 to form cytoophidia in most cells. However, Asn2 alone can rarely form filaments in the absence of Asn1. The discrepancy between Asn1 and Asn2 provides an interesting angle to study the mechanism of the filamentation of metabolic enzymes in general. It would be interested to test if differences in transcription, translation, stability or half-life between Asn1 and Asn2 influence their filamentation outcomes.

Three mutations in the dimerization interface of Asn1 were designed aiming to disrupt the filament formation. We observe no obviously morphological changes of cytoophidia in ASN1^R354E^-GFP ASN2 KO and ASN1^E48K^-GFP ASN2 KO cells, although these two mutant strains show less cells containing cytoophidia than the wild-type strain. The third mutation, *ASN1*^*D330V*^, results in branched cytoophidia, which are hardly seen in the wild-type strain. We also find that *ASN1*^*D330V*^ promotes cytoophidia formation, which reminds us the famous E6V mutation in beta hemoglobin leads to the polymerization of hemoglobin tetramers. Here the reason for Asn1 filamentation may be similar. A new protein-protein interface may be induced by the D330V mutation, either through a stacking or bridging and stacking mechanism [23]. The Asn1 protein level in ASN1^D330V^-GFP ASN2 KO cells is higher than that in ASN1^WT^-GFP ASN2 KO, ASN1^R354E^-GFP ASN2 KO and ASN1^E48K^-GFP ASN2 KO cells. However, the growth rate of ASN1^D330V^-GFP ASN2 KO cells are much slower than the other three strains. Currently we are unable to tell whether branched cytoophidia in ASN1^D330V^-GFP ASN2 KO cells contribute to high protein level and/or slow grow rate. Ultrastructural analysis of cytoophidia containing wild-type and mutant Asn1 proteins will help us gain insights into the molecular mechanism of ASNS cytoophidium assembly.

CTPS cytoophidia was first reported by three studies in 2010 [1–3]. Eight years later, the physiological role of CTPS cytoophidia remains elusive. There were some lines of evidence support that forming filaments can sequester or promote the enzymatic activity of CTPS in various organisms [24–28]. Does the ASNS cytoophidium have similar functions of the CTPS cytoophidium? Since there are quite a few metabolic enzymes showing the filament-forming capability, we speculate that cytoophidia have more general functions in the cell [21, 22]. The close proximity between ASNS and CTPS cytoophidia raises the possibility that both enzymes function coordinately in maintaining metabolic homeostasis in the cytoplasm and nucleus.

In summary, we use budding yeast as a model system to study the filamentation of two glutamine-utilizing enzymes CTPS and ASNS. We have detected the close relationship between CTPS and ASNS cytoophidia both in the cytoplasm and in the nucleus. Moreover, we have demonstrated that two ASNS proteins exhibit differential filament-forming capability even their distribution seems identical in the cytoplasm and nucleus. Finally, we identify a mutation, *ASN1*^*D330V*^, which leads to branched cytoophidia, increased protein level and delayed growth. Our results provide new opportunities to study how filamentation and compartmentation impact glutamine metabolism.

## Materials and methods

### Yeast strains and growth conditions

The yeast strains used in this study are derived from BY4741 [29]. All the *S*. *cerevisiae* strains used are listed in S1 Table. Yeast cells were cultured at 30 °C on YPD rich media (1% Bacto yeast extract, 2% peptone, 2% glucose) or SC medium with appropriate supplements depending on strains. In glucose deprivation assay, exponential phase cells (12 hours culturing) were centrifuged and added into previous media, YP media (1% yeast extract, 2% peptone), distilled water, and 2% glucose solution. After 1 hour 32°C culturing, cells were fixed and pictures were taken by confocal microscope. In glucose recovery assay, 2% glucose was added into culturing system. After 1 hour 32°C culturing, cells were fixed and pictures were taken by confocal microscope.

### Genomic tagging

Fluorescent protein tagging in the genome was done by transforming yeast strains with a PCR product that encoded a selective marker gene and 5’ and 3’ 40-bp flanking sequences homologous to the target gene. pFA6a-mCherry(S65T)-kanMX6 plasmid was updated by pFA6a-GFP(S65T)-kanMX6 [30]. pFA6a-GFP(S65T)-His3MX6 and pFA6a-mCherry(S65T)-KanMX6 were utilized in GFP tagging and mCherry tagging, respectively. The yeast strain BY4741 and plasmids were gifts from Jinqiu Zhou (SIBS, Shanghai, China). PCR products harboring the target gene sequence were used to transform the BY4741 haploid wild type strain.

After transformation and homologous recombination, transformants were selected based on the selective marker. Correct integration results in a C-terminal in-frame GFP fusion, whose expression is driven by the endogenous promoter. Primers used in GFP or mCherry tagging of *ASN1* and *ASN2* are listed in S2 Table. Sequences in upper case indicate homology to the genome of *S*. *cerevisiae* and those in lower case indicate homology to the PCR cassette employed.

### Gene disruption

The ASN1 and ASN2 disruption cassettes were constructed respectively in pRS306 and pRS303 plasmids. For each gene disruption, the 5’ untranslated region and 3’ region were sub-cloned into the corresponding vector, cut using specific restriction enzymes, and then transferred into yeast to replace the given gene with a selective marker by homologous recombination [31]. The disruption cassettes were both linearized with EcoR I. Gene disruption or genomic tagging was confirmed by PCR. Primers used in *ASN1* or *ASN2* deletion are listed in S3 Table.

### Confocal microscopy and stimulated emission depletion microscopy

Yeast cells were spun down for 1 min at 4000 rpm/min. 100 μl of 4% paraformaldehyde were added to each tube to fix the cells for 10 min at room temperature, and then the cells were washed once with PBS.

A few microliters of cell suspension were mixed with agarose gel (1.2% low melting temperature agarose and 100 μM N-propyl gallate in YPD) to avoid movement of the cells during imaging. Images were acquired under 63x objectives on a Zeiss LSM 710 inverted fluorescence confocal microscope. For the co-localization experiment, super-resolution stimulated emission depletion (STED) microscopy was used. The abundance of filaments in cells was quantified by capturing images in at least four different areas containing a minimum of 100 cells each.

### Homology modeling of *S*. *cerevisiae* Asn1

The homology model of Asn1 of *S*. *cerevisiae* and its dimer were built based on the structure of *E. coli* ASNB [20] (PDB: 1CT9), by using the MODELLER software [32]. The sequence identity between Asn1 and ASNB is 47%. Residues on the dimer interfaces are highly conserved.

### Mutagenesis

This method is applied to alter single nucleotides in DNA that has been previously cloned in a plasmid. The plasmid is then transferred back into yeast cells to generate a strain that carries the mutated version of the gene of interest on the plasmid but lacks the corresponding wild type locus in the genome in order to assess the function of the mutant allele. Insert fragments (including about 1 kb upstream of the promoter element and 0.5 kb downstream of the 3′-untranslated region of GFP-tagged target gene) were cloned into pRS315. Subsequently, this vector was transformed into an *ASN1* deletion strain. This strain was regarded as the control strain. The vector was also used as the template for point mutant vectors (E48K, D330V and R354E). DpnI was used to eliminate the template and then point mutant vectors were transformed into the *ASN1* deletion strain. Oligonucleotide primers used for site-directed mutagenesis are listed in S4 Table.

### Protein abundance of Asn1 or mutant Asn1 (E48K, D330V and R354E)

We used Western blots to analyze the protein level of ASNS. Yeast cells were collected at different time points and run on an 8% SDS-PAGE gel before transfer to a PVDF membrane (Bio-Rad). Following incubation in blocking buffer (TBST containing 5% nonfat dry milk (Bio-Rad)) for 1 h, the membrane was hybridized in blocking buffer containing primary antibody overnight at 4 °C. The membrane was then washed and incubated with a horseradish peroxidase-conjugated secondary antibody for 1 h at room temperature. Detailed information about the antibodies used, with concentration and source, can be found in S5 Table. Enhanced ECL was used for signal detection. The image signal was collected by an Amersham Imager 600 (GE Healthcare Life Science). Data were processed and analyzed by Image J software. All quantifications were done by normalizing protein levels to actin.

### Spot assay of yeast cells

Yeast cells were grown overnight and diluted roughly to an optical density (O.D.) of 1.0. Four or five ten-fold dilutions were spotted onto YPD agar plates. A multichannel pipette was used to transfer 10 μl cells to 90 μl culture medium. After mixing well, 8 μl of each dilution were spotted onto the plates. Plates were incubated at 30 °C, examined regularly, and photographed after 48 hours incubation.

## Supporting information

S1 Table *S*. *cerevisiae* strains used in this study. The strain name, number and genotype of strains used in our study are listed in this table. All strains are haploid and derive from the wild-type haploid (BY4741). The introduced plasmid carries the wild type, mutational version of Asn1 and the corresponding wild-type locus in the genome is disrupted.
(TIF)

S2 Table Primers used for genomic tagging. Genomic tagging with fluorescent protein is based on the homologous recombination. Sequence homology to pFA6a-GFP(S65T)-His3MX6 for GFP tagging are indicated small letter and in bold. Sequence homology to pFA6a-mCherry(S65T)-KanMX6 for mCherry tagging are indicated small letter and underlined.
(TIF)

S3 Table Primers used for gene disruption. Gene disruption is based on the fact that linear DNA fragments carrying a selectable marker gene with homology regions to an interest gene. 5’ and 3’ untranslated region of *ASN1* are amplify by ASN1 UHA-F, ASN1 UHA-R and ASN1 DHA-F, ASN1 DHA-R primers respectively. ASN1 KO testing F and ASN1 KO testing R primers are used to confirm the genotype. ASN2 disruption strain is obtained by similar approach.
(TIF)

S4 Table Oligonucleotide primers used for site-directed mutagenesis. Upstream of the promoter element and coding sequence of *ASN1-GFP* with selective marker are amplify by A1-UF and A1-UR primers. Downstream of the 3’—untranslated region of ASN1 is amplified by A1-DF and A1-DR primers. For point mutant vectors (E48K, D330V, R354E), corresponding primers are used. Point mutations are indicated in bold and underlined.
(TIF)

S5 Table Antibodies used with concentration and source. We analyze the protein level of ASNS by detecting GFP. In this table, we provide the information of antibody used with concentration and source.
(TIF)
