# Supplementary figures and images for: Filamentation of asparagine synthetase in Saccharomyces cerevisiae
Source: PLoS Genet. 2018 Oct 26;14(10):e1007737. doi: 10.1371/journal.pgen.1007737 (PMC6221361; doi:10.1371/journal.pgen.1007737)

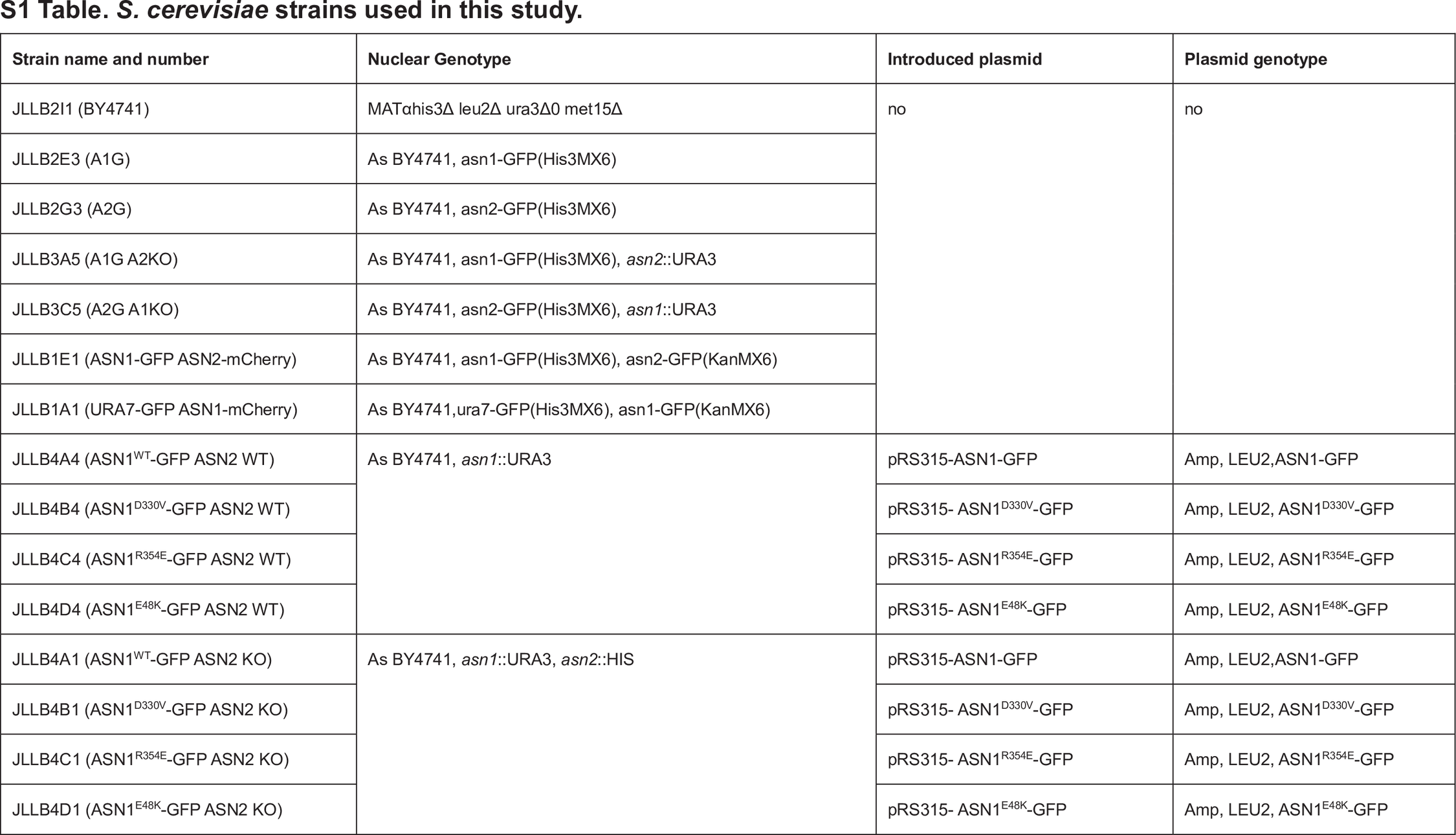

Supplement: S1 Table — The strain name, number and genotype of strains used in our study are listed in this table. All strains are haploid and derive from the wild-type haploid (BY4741). The introduced plasmid carries the wild type, mutational version of Asn1 and the corresponding wild-type locus in the genome is disrupted. (TIF) [file pgen.1007737.s001.tif]

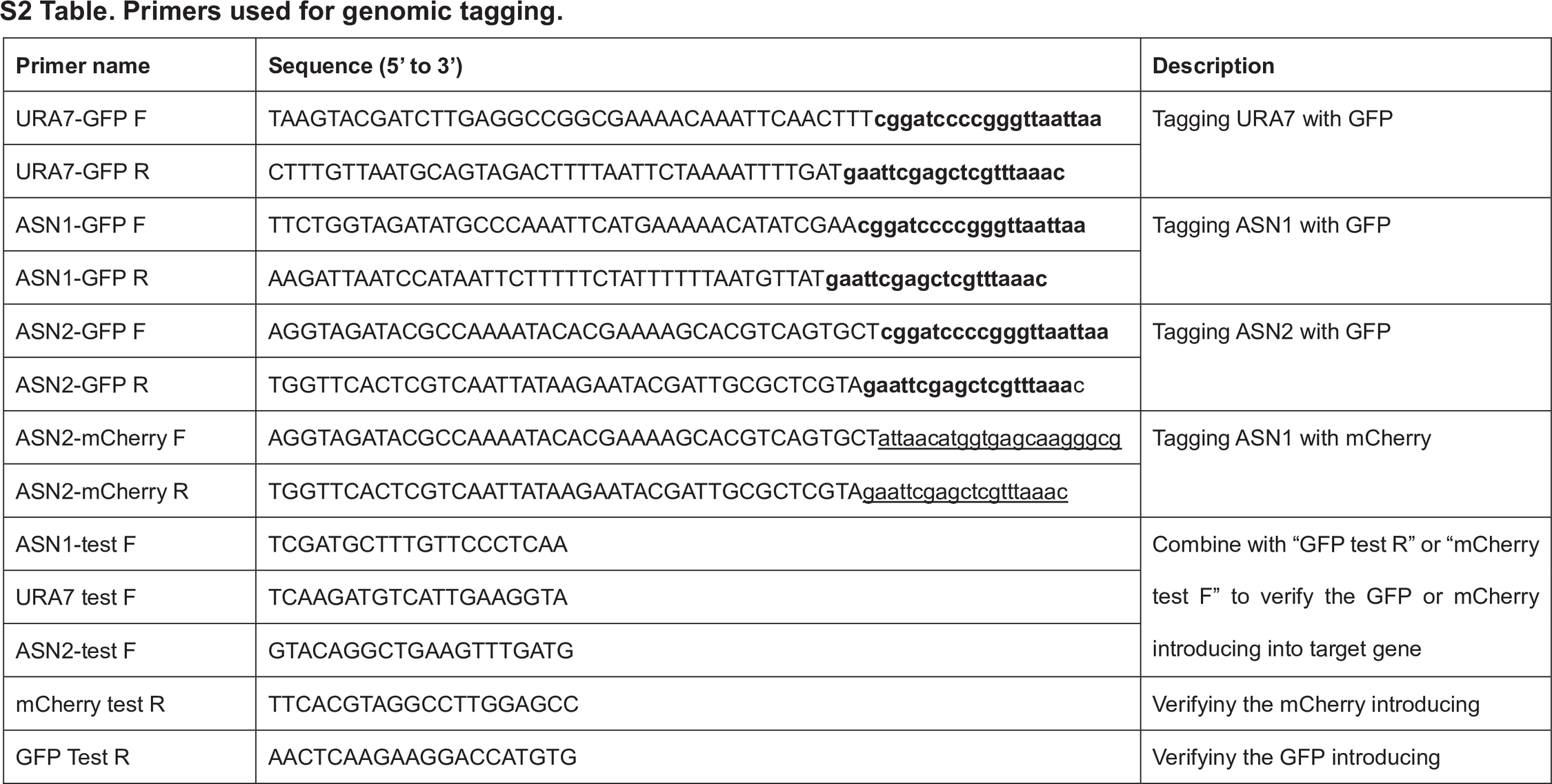

Supplement: S2 Table — Genomic tagging with fluorescent protein is based on the homologous recombination. Sequence homology to pFA6a-GFP(S65T)-His3MX6 for GFP tagging are indicated small letter and in bold. Sequence homology to pFA6a-mCherry(S65T)-KanMX6 for mCherry tagging are indicated small letter and underlined. (TIF) [file pgen.1007737.s002.tif]

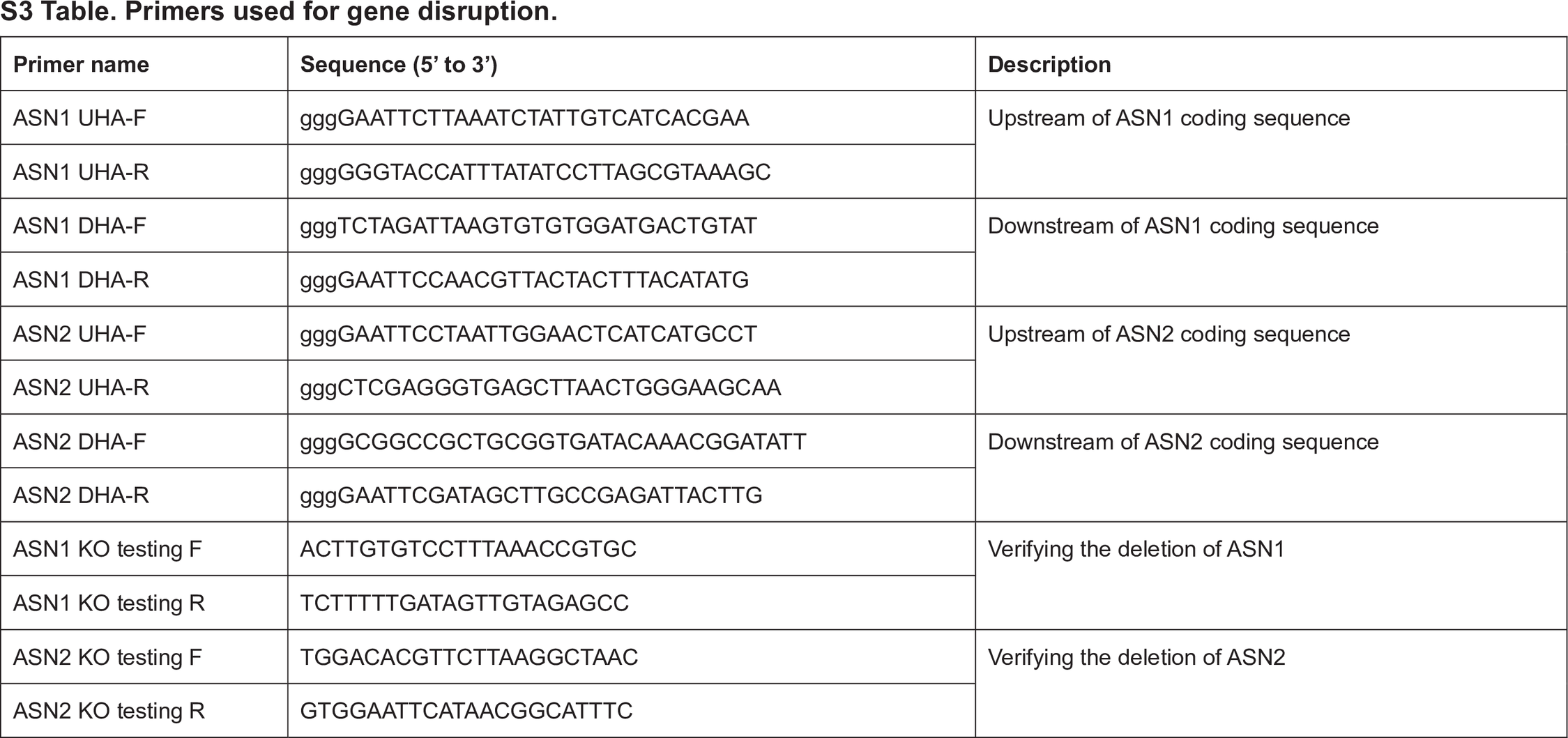

Supplement: S3 Table — Gene disruption is based on the fact that linear DNA fragments carrying a selectable marker gene with homology regions to an interest gene. 5’ and 3’ untranslated region of ASN1 are amplify by ASN1 UHA-F, ASN1 UHA-R and ASN1 DHA-F, ASN1 DHA-R primers respectively. ASN1 KO testing F and ASN1 KO testing R primers are used to confirm the genotype. ASN2 disruption strain is obtained by similar approach. (TIF) [file pgen.1007737.s003.tif]

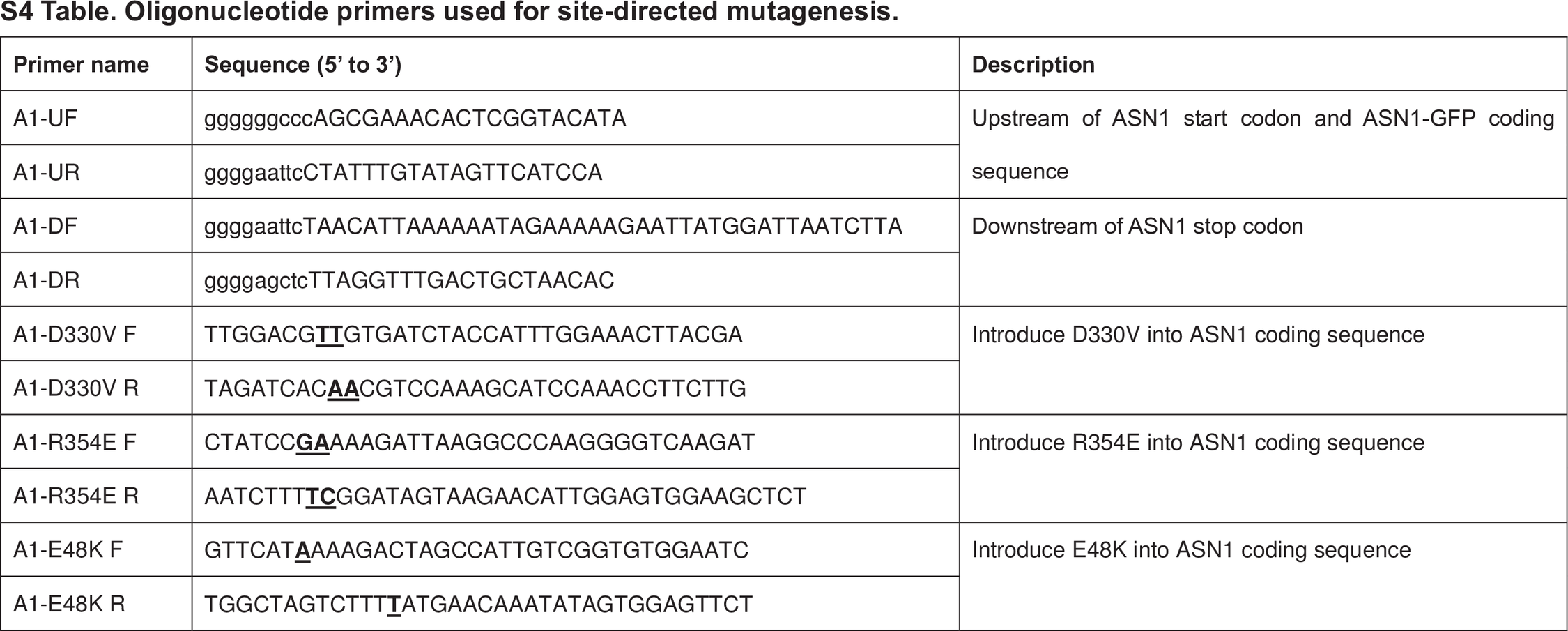

Supplement: S4 Table — Upstream of the promoter element and coding sequence of ASN1-GFP with selective marker are amplify by A1-UF and A1-UR primers. Downstream of the 3’—untranslated region of ASN1 is amplified by A1-DF and A1-DR primers. For point mutant vectors (E48K, D330V, R354E), corresponding primers are used. Point mutations are indicated in bold and underlined. (TIF) [file pgen.1007737.s004.tif]

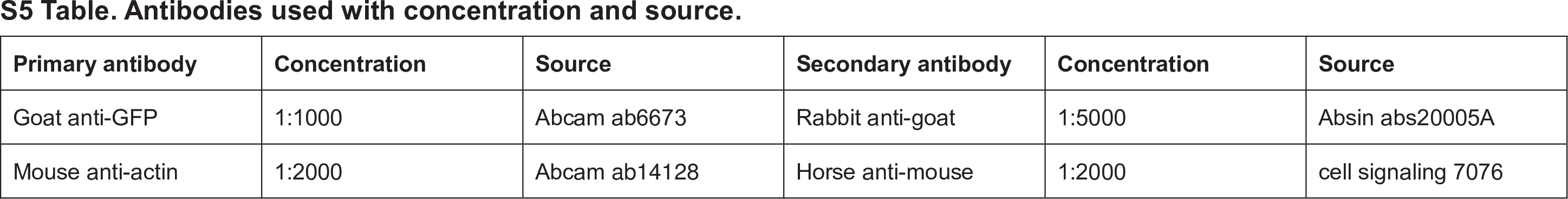

Supplement: S5 Table — We analyze the protein level of ASNS by detecting GFP. In this table, we provide the information of antibody used with concentration and source. (TIF) [file pgen.1007737.s005.tif]
